# Dynamic-related protein 1 inhibitor eases epileptic seizures and can regulate equilibrative nucleoside transporter 1 expression

**DOI:** 10.1186/s12883-020-01921-y

**Published:** 2020-09-22

**Authors:** Zhong Luo, Jing Wang, Shirong Tang, Yongsu Zheng, Xuejiao Zhou, Fei Tian, Zucai Xu

**Affiliations:** 1 Department of Neurology, The Affiliated Hospital of Zunyi Medical University, 149 Dalian Road, Zunyi, 563003 Guizhou China; 2 Department of Neurology, The Thirteenth People’s Hospital of Chongqing, Chongqing, 400053 China; 3 Department of Neurology, Xuanwu Hospital, Capital Medical University, Beijing, 100053 China

**Keywords:** Epileptic seizure, Drp1, ENT1, Mitochondria, SE

## Abstract

**Background:**

Dynamic-related protein 1 (Drp1) is a key protein involved in the regulation of mitochondrial fission, and it could affect the dynamic balance of mitochondria and appears to be protective against neuronal injury in epileptic seizures. Equilibrative nucleoside transporter 1 (ENT1) is expressed and functional in the mitochondrial membrane that equilibrates adenosine concentration across membranes. Whether Drp1 participates in the pathogenesis of epileptic seizures via regulating function of ENT1 remains unclear.

**Methods:**

In the present study, we used pilocarpine to induce status epilepticus (SE) in rats, and we used mitochondrial division inhibitor 1 (Mdivi-1), a selective inhibitor to Drp1, to suppress mitochondrial fission in pilocarpine-induced SE model. Mdivi-1administered by intraperitoneal injection before SE induction, and the latency to firstepileptic seizure and the number of epileptic seizures was thereafter observed. The distribution of Drp1 was detected by immunofluorescence, and the expression patterns of Drp1 and ENT1 were detected by Western blot. Furthermore, the mitochondrial ultrastructure of neurons in the hippocampal CA1 region was observed by transmission electron microscopy.

**Results:**

We found that Drp1 was expressed mainly in neurons and Drp1 expression was significantly upregulated in the hippocampal and temporal neocortex tissues at 6 h and 24 h after induction of SE. Mitochondrial fission inhibitor 1 attenuated epileptic seizures after induction of SE, reduced mitochondrial damage and ENT1 expression.

**Conclusions:**

These data indicate that Drp1 is upregulated in hippocampus and temporal neocortex after pilocarpine-induced SE and the inhibition of Drp1 may lead to potential therapeutic target for SE by regulating ENT1 after pilocarpine-induced SE.

## Background

Status epilepticus (SE) is an especially common and life-threatening neurological emergency characterized by abnormally prolonged epileptic seizures. Although several neurological dysfunctions in the hippocampus have been reported after pilocarpine-induced SE [1], essential pathophysiologic mechanisms are still not fully appreciated. It is, therefore, of paramount importance to explore the underlying mechanism and provide new therapeutic targets for SE.

Mitochondria are virtually ubiquitous in the cytoplasm and play a vital role in cell metabolism, being involved in processes such as adenosine triphosphate (ATP) generation, which provides energy for cell activities, antioxidant stress, calcium homeostasis, and the synthesis of neurotransmitters in specific neuron types [2]. Epileptic seizure can induce glutamate overexposure, overactivation of NMDA, and AMPA/KA receptors, increasing Ca2+ influx into neurons [3]. This Ca2+ can be taken up by mitochondria and lead to mitochondrial calcium overload [4] and oxidative stress, consequently damaging the mitochondria and neurons in kainic acid (KA), pilocarpine, and pentylenetetrazole (PTZ) treatment models [5]. During epileptic seizures, neurons have a soaring demand for energy, and the enzyme protein synthesis of the respiratory chain in mitochondria increases, which leads to mitochondrial protein synthesis functional compensatory hyperactivity. When ATP synthesized by mitochondria is insufficient to meet the requirements of neuron activity, abnormal functions and even necrosis of neurons will occur. Long-term epileptic seizures can damage mitochondria, causing decreased mitochondrial membrane potential, increased mitochondrial permeability, and the release of cytochrome C (Cyt C) and apoptosis inducing factor (AIF) into the cytoplasm, which induce apoptosis and increase sensitivity to epileptic damage [6]. Mitochondria represent the primary site of reactive oxygen species (ROS) production, and mitochondrial ROS contribute to neuronal damage in seizures and play a vital role in the pathogenesis of epilepsy [7, 8]. For example, Zhang et al. demonstrated that succinate accumulation plays a vital role in mitochondrial ROS production, oxidative stress, mitoSOX levels, and seizure phenotypes in the KA-induced SE rat model [9].

Drp1, a key protein that precisely regulates mitochondrial fission, is highly expressed in brain and heart, and its abnormal expression can lead to the formation of various diseases. Studies have shown that Drp1 is closely related to mitochondrial-dependent apoptosis of hippocampal neurons in pilocarpine-induced epileptic rats, and inhibition of mitochondrial fission by Drp1 selective inhibitors has an obvious protective effect against neuronal injury in epileptic seizures [10, 11]. After epileptic seizures in animal models, mitochondria are damaged, and Drp1-mediated mitochondrial fission and neuron apoptotic signals are stimulated by increasing mitochondrial outer membrane permeability [12]. In the KA-induced SE model, mitophagy and neuronal degeneration are increased in the hippocampus [9]. Mitophagy plays a key role in controlling mitochondrial quality by removing damaged or redundant mitochondria [13, 14]. Hwajin Kim et al. found that Mdivi-1, a specific inhibitor of mitochondrial division protein Drp1 by inhibiting Drp1 self-assembly, prevents Parkin-mediated mitophagy by inhibiting the expression of Hsp72 in mitochondria isolated from the hippocampus of KA-induced seizure mice [15]. Equilibrative nucleoside transporter 1 (ENT1) can affect receptors activities on the neuron surface by regulating the concentration of adenosine, thus regulating cell signal transduction [16, 17]. Adenosine is generated mainly by the decomposition of ATP, and Drp1-mediated mitochondrial fission dysfunction can increase the synthesis of ATP and lead to the intra- and extracellular adenosine imbalance, which can induce hyperexcitability of neurons by ENT1 [18]. Therefore, we hypothesize that inhibition of Drp1 by Mdivi-1 may attenuate epileptic seizure severity by regulating the function of ENT1.

Thus, in the present study, we used pilocarpine to induce SE in rats. We determined the endogenous location and alteration of Drp1 in the hippocampus and temporal neocortex using Western blot and immunofluorescence analyses after SE. Additionally, we used Mdivi-1 to suppress mitochondrial division in vivo in the pilocarpine-induced SE model of rats. We aimed to investigate the alteration of mitochondrial division after SE, and we evaluated the role of Drp1 against mitochondrial damage and ENT1 expression.

## Methods

### Animals and induction of SE

All experimental protocols were reviewed and approved by the Commission of Zunyi Medical University for Ethics of Experiments on Animals. Adult male SD rats weighing 180–220 g (6–8 weeks) were obtained from the experimental animal center of the Third Military Medical University [19]. All animals were maintained in a temperature and humidity-controlled room with free access to food and tap water. The experiments were conducted in accordance with the National Institutes of Health Guide for the Care and Use of Laboratory Animals, based on the guidelines of the World Medical Association Declaration of Helsinki. Additionally, all efforts were made to minimize animal numbers and suffering. To induce the lithium-pilocarpine model of SE, rats were pretreated intraperitoneally with lithium chloride (3 mEq/kg, Sigma USA) 18 h before atropine sulfate injection (1 mg/kg, i.p., Sigma USA) and 30 min after atropine sulfate injection. Rats in the experimental group were injected with pilocarpine (30 mg/kg, i.p.; Sigma) [20, 21], and rats in the control group received the same treatment (both lithium chloride and atropine sulfate), except that saline was used instead of pilocarpine. Only those rats exhibiting sustained severe SE with convulsive seizures (Class IV-V) were included for further studies [22]. Epileptic seizures were terminated by an intraperitoneal (i.p.) administration of diazepam (10 mg/kg) 60 min after the onset of convulsive seizures [10].

After 1 week of adjustment to their environment, rats were randomly assigned to groups based on different treatments: (1) pilocarpine group (pilo): lithium chloride, atropine sulfate, and pilocarpine administered successively; (2) DMSO group (pilo + DMSO): DMSO (0.1%) injected intraperitoneally 30 min before pilocarpine injection; (3) pilocarpine and Mdivi-1 group (pilo + Mdivi-1): Mdivi-1(1.2 mg/kg, ENZO USA) injected intraperitoneally 30 min before pilocarpine injection. The solvent for Mdivi-1 was DMSO (25 mg/ml). For behavioral assessment, the intensity of epileptic seizures was evaluated by Racine’s scale, and the latency to the first onset of Class IV epileptic seizure was recorded [23].

### Rat brain tissue preparation

Rats were sacrificed by decapitation at 6, 24, and 72 h and at 1 week after the onset of SE. Euthanization was accomplished humanely by trained laboratory personnel via an intraperitoneal injection of saline-diluted Euthasol (150 mg/kg) containing pentobarbital sodium (390 mg/mL) and phenytoin sodium (50 mg/mL), followed by cervical dislocation at different experimental timepoints [24]. For western blot analysis, the rats were anesthetized (i.p., saline-diluted Euthasol (150 mg/kg) containing pentobarbital sodium (390 mg/mL) and phenytoin sodium (50 mg/mL))and decapitated, and the bilateral temporal cortices and the hippocampus were immediately removed on ice for further protein extraction. For histological analyses, brains were post-fixed with 4% paraformaldehyde (PFA) in PBS overnight at 4 °C after perfusion with PBS, and then sectioned using a cryostat. For electron microscopy analysis, the rats were anesthetized and decapitated after saline infusion, followed by rapid separation of the hippocampal CA1 area on ice. The hippocampal section was then cut into 1-mm^3^ pieces and fixed in 4% glutaraldehyde for 2 h at 4 °C [25, 26].

### Western blotting

Western blotting was performed as previously described [26]. Temporal cortex and hippocampal tissues were removed and treated with 1.5 M radioimmune precipitation buffer (RIPA) and PMSF. The protein concentrations of all samples were determined using the Enhanced BCA Protein Assay Kit (Beyotime, China). Equal amounts of total protein (30 micrograms) were separated by 10% sodium dodecyl sulfate polyacrylamide gel electrophoresis (SDS-PAGE) and then transferred to polyvinylidene difluoride (PVDF, Millipore, USA) membranes. The membranes were sequentially blocked with Blocking Reagent (Beijing Dingguo Biotechnology, China) at room temperature for 30 min and then incubated with primary antibodies as follows: anti-Drp1 (1:500 ab184247, Abcam, USA) and β-actin antibody (1:1000, Santa Cruz, USA) at 4 °C overnight. The next day, the membranes were washed three times with TBST (10 min at a time) and then incubated with secondary antibody (goat anti-rabbit IgG HRP, 1:1000, Santa Cruz, USA) at room temperature for 1 h. After washing three times with TBST, the bands were visualized using SuperSignal West Pico Chemiluminescent HRP substrate (Rockford, USA), and quantity one software (Bio-Rad, USA) was used to quantify the density of the bands.

### Immunohistochemical staining and hematoxylin and eosin staining

The sections of rat brain were dewaxed using biological transparent agent wax. Antigen retrieval was performed for 20 min in citrate solution (0.01 mol/L, pH 6.0). Next, the sections were equilibrated at room temperature for 30 min and rinsed three times with 0.1 M PBS (10 min per time). Endogenous peroxidase was removed with 3% H_2_O_2_ in ethanol for 10 min at room temperature. The sections were rinsed three times with 0.1 M PBS and then blocked in blocking buffer (containing 5% BSA in PBS) for 30 min at 37 °C. The sections were incubated with primary antibody (Drp1, 1:500, Abcam, USA) overnight in a wet box at 4 °C. Sequentially, the sections were rinsed three times with PBS and incubated with biotinylated secondary antibody at room temperature for 1 h. After three rinses with PBS, diaminobenzidine (DAB) was used for color development, and the sections were mounted with coverslips. Finally, the images were captured by optical microscopy (Nikon, Japan).

After pretreatment, the rats in each group were sacrificed by cervical dissection to obtain the brain tissues. The specimens were fixed in 4% PFA at room temperature for 24 h and embedded in paraffin. After dewaxing with xylene, the paraffin-embedded slices were stained with hematoxylin and eosin (HE), dried with 1% hydrochloric acid in alcohol, and mounted with neutral balata and coverslips. The slides were viewed by optical microscopy. In each section, the hippocampal CA3 region was examined to observe neuron damage, and positive cells were counted and photographed.

### Transmission electron microscopy

The brain was fixed in 2.5% glutaraldehyde solution. After the brain tissue was fully fixed, the CA1 area of the hippocampus was cut to obtain 1-mm^3^ specimens. The specimens were placed in fixative solution (fixed for more than 24 h) and preserved for transmission electron microscopy. After rinsing with PBS (3 times, 15 min each), the specimens were fixed in 2% osmium tetroxide for 2 h and then rinsed again with PBS (3 times, 15 min each). The samples were placed in alcohol for gradient dehydration, immersed in 90% acetone at 4 °C for 20 min, and then immersed in 100% acetone for 15 min. The specimens were then placed in embedding fluid, after which they were cut with a special ultra-thin microtome for electron microscopy (60 nm). The prepared aluminum citrate and uranyl acetate were used for dyeing. After obtaining photos with a projection electron microscope, image-pro Plus 6.0 software was used for Image processing and data analysis. Hippocampal CA1 neurons were identified based on their characteristic nuclei as previously described [27]. For each sample, 10 fields of view were selected at random for observation by electron microscopy, and the images were captured using a Gatan-780 CCD camera.

### Immunofluorescence staining

Frozen sections were selected at random and permeabilized with 0.1% Triton X-100 in PBS. The sections were blocked with blocking buffer (containing 5% goat serum, 2% BSA, and 0.1% Triton X-100 in PBS, pH 7.4) for 1 h at room temperature. Then, the sections were incubated with a mixture of primary antibody buffer containing a Drp1 antibody (1:500, Abcam, USA), microtubule-associated protein 2 (MAP 2) antibody (1:500, Sysy, Germany), and glial fibrillary acidic protein (GFAP) antibody (1:200, Boster, China) overnight at 4 °C. The next day, after rinsing three times with PBST, the sections were incubated with a mixture of fluorochrome-conjugated secondary antibody buffer containing Alexa Fluor 488-conjugated antibody (1:100), DyLight 594-conjugated antibody (1:100), and Alexa Fluor 405-conjugated antibody (1:100) in the dark at room temperature for 2 h. The sections were then washed with PBS in the dark, mounted with 50% glycerol, and cover slipped. The images were captured by laser scanning confocal microscopy (Leica).

### Statistical analysis

All data are presented as means and SEM (standard error of the mean), and statistical analysis were conducted using SPSS version 17. Data between groups were analyzed using one-way analysis of variance (ANOVA). A *p* value < 0.05 was considered statistically different.

## Results

### Effects of status epilepticus on Drp1 expression in brain tissues

**Fig. 1:**
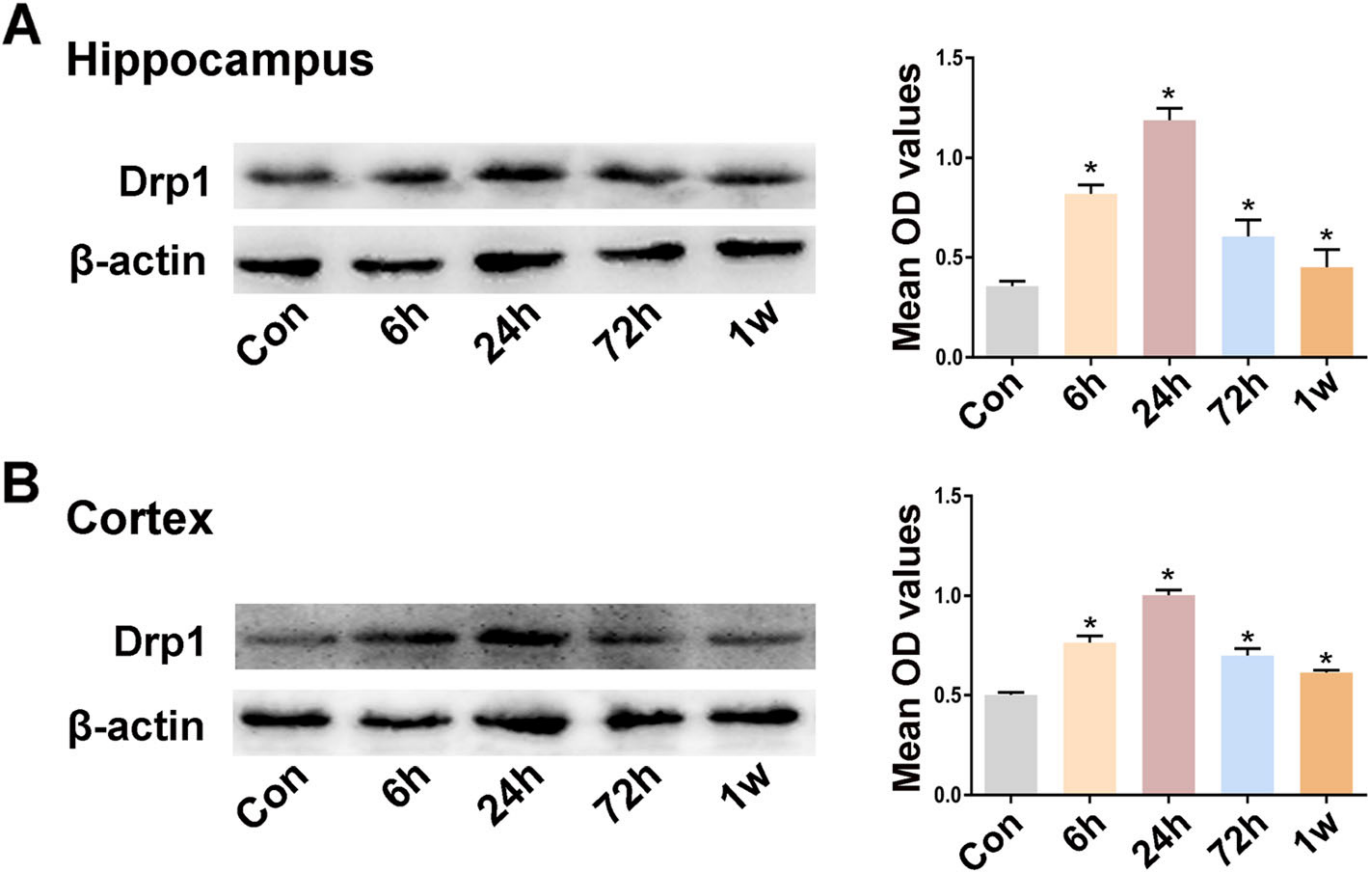
Expression of Drp1 in brain tissues of pilocarpine-induced epileptic rats. **a**-**b** Representative images of the expression of Drp1 in the hippocampus and cortex from epileptic rats (*n* = 5 per group) and control mice (*n* = 5 per group) and quantification of immunoblots. Data are presented as means ± SEM and are representative of at least 3 independent repeats. *P < 0.05 vs Con, by one-way ANOVA followed by the LSD-*t* test

To clearly determine Drp1 expression alterations in response to SE, we used the pilocarpine-induced SE model. The Drp1 expression levels were quantified by Western blotting of brain tissues from control and lithium-pilocarpine-treated rats at different time points (6, 24, and 72 h and 1 week post-SE). Drp1 was expressed at basal levels in hippocampal tissues from the control group, and the immunoblot density ratio of Drp1 to β-actin was 0.45 ± 0.05. After SE onset, the immunoblot density ratios of Drp1 to β-actin were 0.76 ± 0.07, 1.17 ± 0.05, 0.75 ± 0.02, and 0.61 ± 0.02 at different time points (6, 24, and 72 h and 1 week post-SE, *P* > 0.05). Notably, the results revealed an up-regulation of Drp1 expression relative to control conditions in lithium-pilocarpine-treated SE groups, and Drp1 expression levels peaked at 24 h. Drp1 expression increased and remained elevated until 1 week after epileptic seizure onset (Fig. 1). This increase was observed in both the hippocampus and neocortex.

### Immunofluorescence labeling revealed Drp1 localization in the epileptic rat hippocampus

**Fig. 2:**
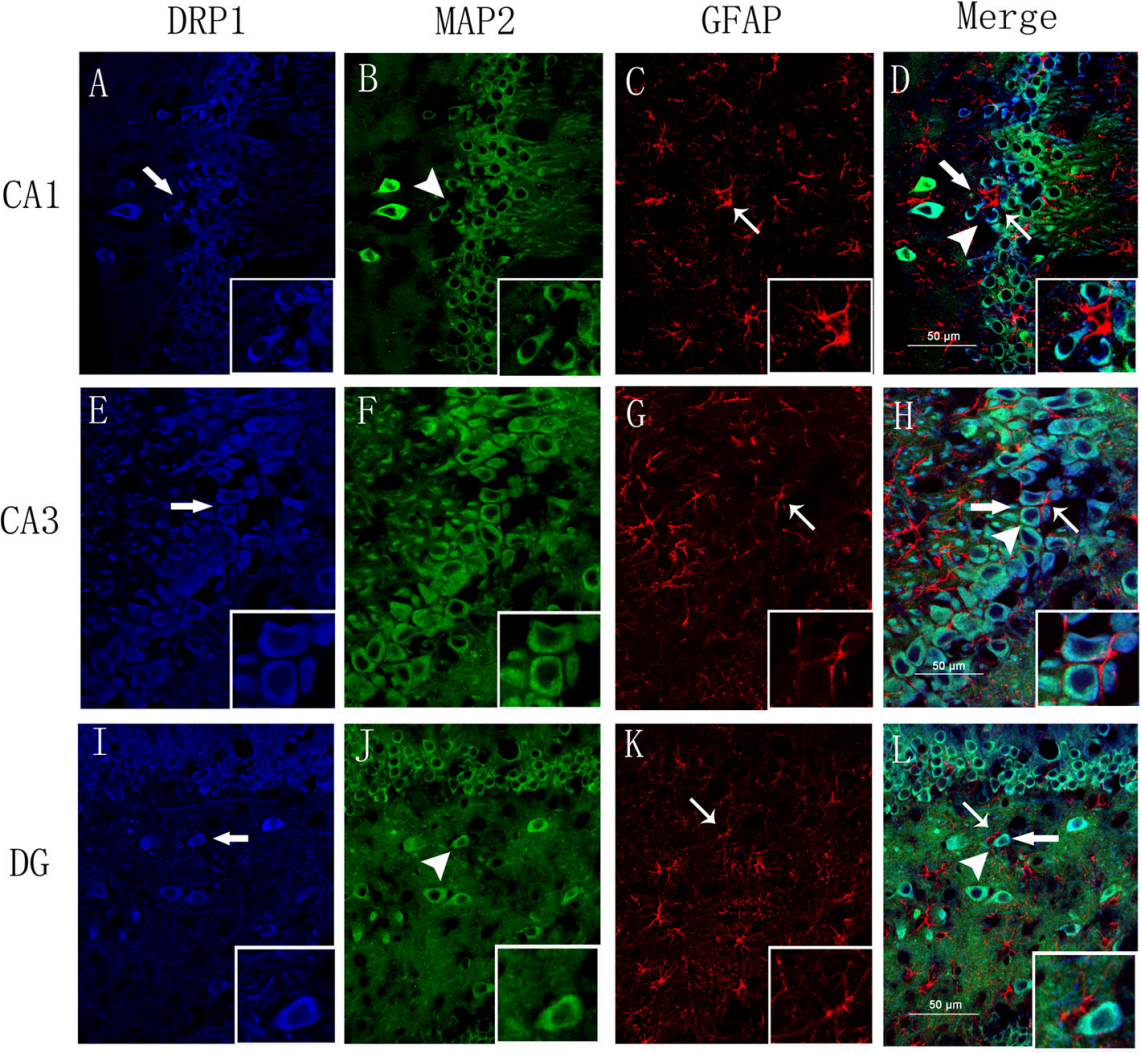
Localization of Drp1 in hippocampal CA1, CA3, and hilus regions of epileptic rats. **a**-**d**, **e**-**h** and **i**-**l** In hippocampal CA1, CA3, and hilus regions from the pilocarpine-induced epileptic rat model, Drp1 colocalized with MAP 2. Scale: 50 μm

Immunofluorescence staining was used to further refine the localization of Drp1 in rat brain 24 h after SE. The localization of Drp1 in brain sections of pilocarpine-treated rats was estimated using Drp1, neuronal MAP 2, and astrocyte marker GFAP antibodies. We found that Drp1 (blue) was coexpressed with MAP 2 (green) in neurons but not with GFAP (red) in astrocytes in the CA1, CA3, and hilus regions of the hippocampus (Fig. 2).

### Effects of Mdivi-1 on epileptic seizure activity

**Fig. 3:**
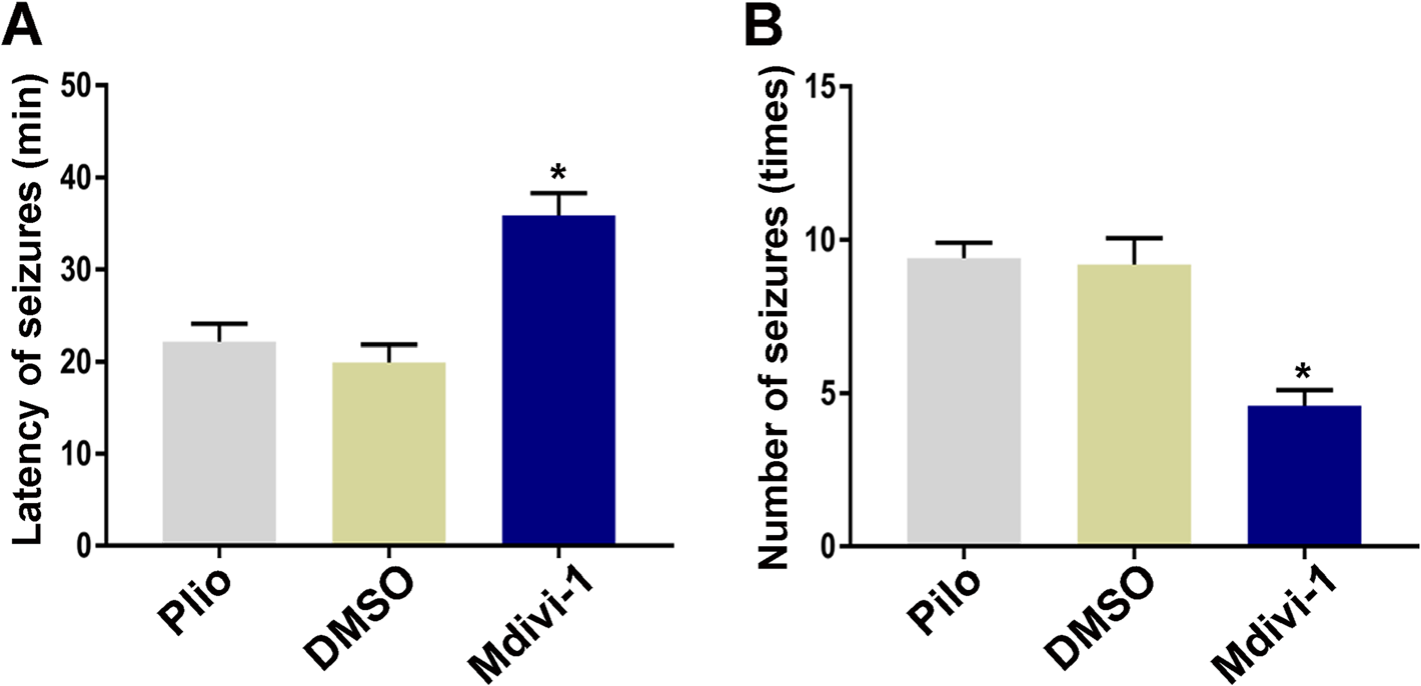
Drp1 modulates the latency of first epileptic seizure and the number of epileptic seizures after pilocarpine injection. **a** and **b** Quantitative analysis of the latency of epileptic seizures and the total number of epileptic seizures in pilocarpine-induced epileptic rats with different treatments. Data are presented as means ± SEM, *n* = 5 per group. *P < 0.05 compared with the DMSO group, by one-way ANOVA followed by the LSD-*t* test

To test whether Drp1 could interfere with the epileptic seizure phenotype, we measured the effect of the Drp1 antagonist Mdivi-1 on epileptic seizure activities. Mdivi-1 (1.2 mg/kg, i.p.) was intraperitoneally administered 30 min before the induction of epileptic seizures with pilocarpine. The latencies of the first epileptic seizure in the pilo + DMSO group, pilo + Mdivi-1 group and pilo group were 24.4 ± 5.12, 38.8 ± 1.30, and 23.6 ± 4.67 min, respectively. Differences in latency were statistically significant. Mdivi-1 increased the epileptic seizure latency in pilocarpine-treated rats. In terms of epileptic seizure severity, the numbers of epileptic seizures within 1 h of the pilo group, pilo + Mdivi-1 group, and pilo + DMSO group were 9.0 ± 0.52, 4.5 ± 0.33, and 9.0 ± 0.37, respectively. No significant differences in latencies or numbers of epileptic seizures in rats were found between pilo and pilo + DMSO (*P* > 0.05). Compared with pilo + DMSO, the latency in the pilo + Mdivi-1 group was significantly extended after pilocarpine injection, and the number of epileptic seizures was significantly reduced. Intraperitoneal injection of Mdivi-1 significantly extended the latency and reduced the number of epileptic seizures in pilocarpine-induced epileptic rats (*P* < 0.05); however, the effect of 0.2% DMSO on these parameters was not statistically significant (Fig. 3).

### Effects of Mdivi-1 on the expression of Drp1 in brain tissues

**Fig. 4:**
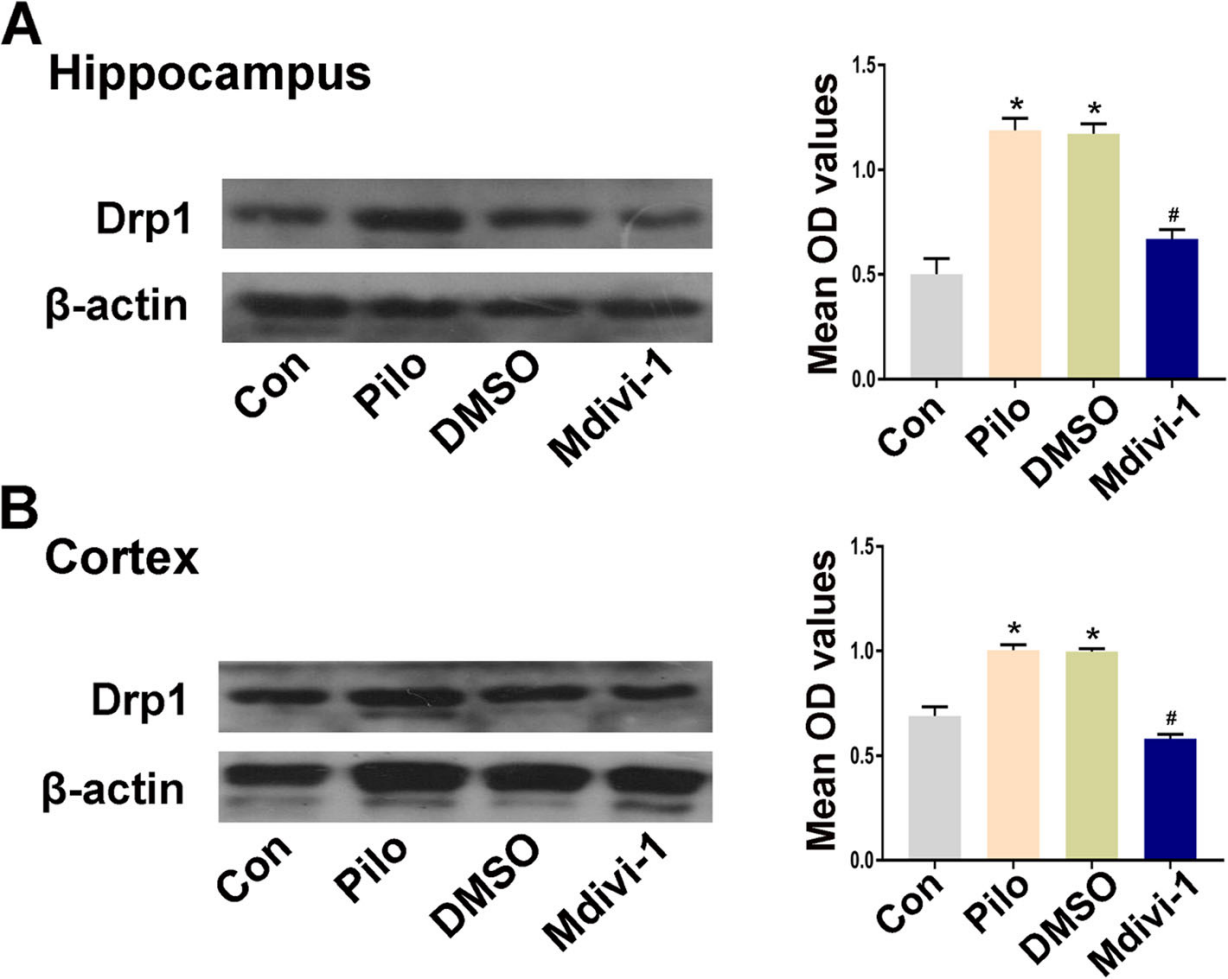
Effects of Mdivi-1 on the expression of Drp1 in brain tissue of pilocarpine-induced epileptic rats. **a**-**b** Representative images of the expression of Drp1 in the hippocampus and cortex from different treatment groups (*n* = 5 per group) and quantification of immunoblots. Data are presented as means ± SEM and are representative of at least 3 independent repeats. *P < 0.05 compared with the Con group, #*P* < 0.05 compared with the pilo + DMSO group, by one-way ANOVA followed by the LSD-*t* test

According to these results, the highest expression level of Drp1 occurred 24 h after the onset of epileptic seizures. Therefore, 24 h after epileptic seizure onset was selected as the time point for measuring the effects of the Drp1 inhibitor Mdivi-1, which was injected intraperitoneally 30 min before the epileptic seizure model was induced. In the rat hippocampus, the immunoblot density ratios of Drp1 to β-actin in the pilo + Mdivi-1 and pilo + DMSO group were 0.58 ± 0.04 and 0.99 ± 0.03, respectively. Western blot analysis showed that the expression level of Drp1 in the pilo + Mdivi-1 group was significantly lower than in the pilo + DMSO group at 24 h after epileptic seizure onset (*P* < 0.05). No significant difference was found in the expression of Drp1 between the pilo + DMSO and the pilo group 24 h after epileptic seizure onset (*P* > 0.05) (Fig. 4).

### Effect of Mdivi-1 on hippocampal Drp1 expression measured by immunohistochemistry

**Fig. 5:**
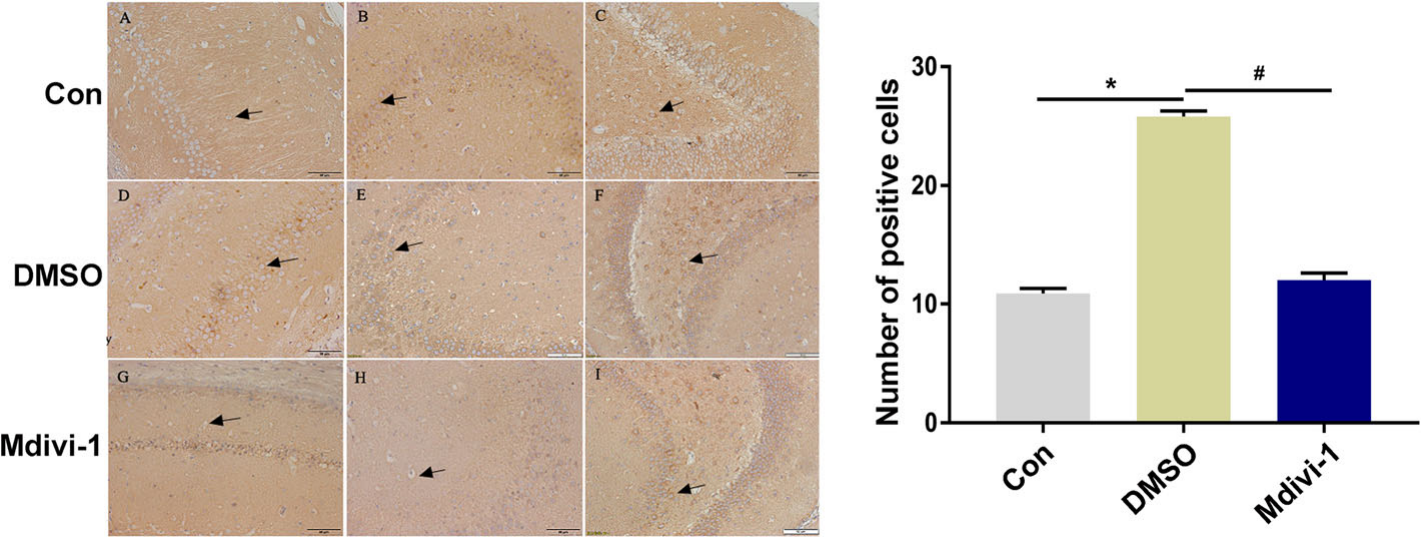
Immunohistochemical detection of Drp1 expression in the hippocampus of different treatment groups. **a**-**i** Representative images of immunohistochemical staining for Drp1 in the hippocampus tissues from different treatment groups and quantification of positive cells. Data are presented as means ± SEM and are representative of at least 3 independent repeats, *n* = 9 sites per group. **a**, **d**, and **g** show the CA1 hippocampal area; **b**, **e**, and **h** show the CA3 hippocampal area; and **c**, **f**, and **i** show the hilus hippocampal area. The plot shows the quantification of A-I. *P<0.05 compared with the con group, ^*#*^*P*<0.05 compared with the pilo + DMSO group. Scale: 50 μm

**Fig. 6:**
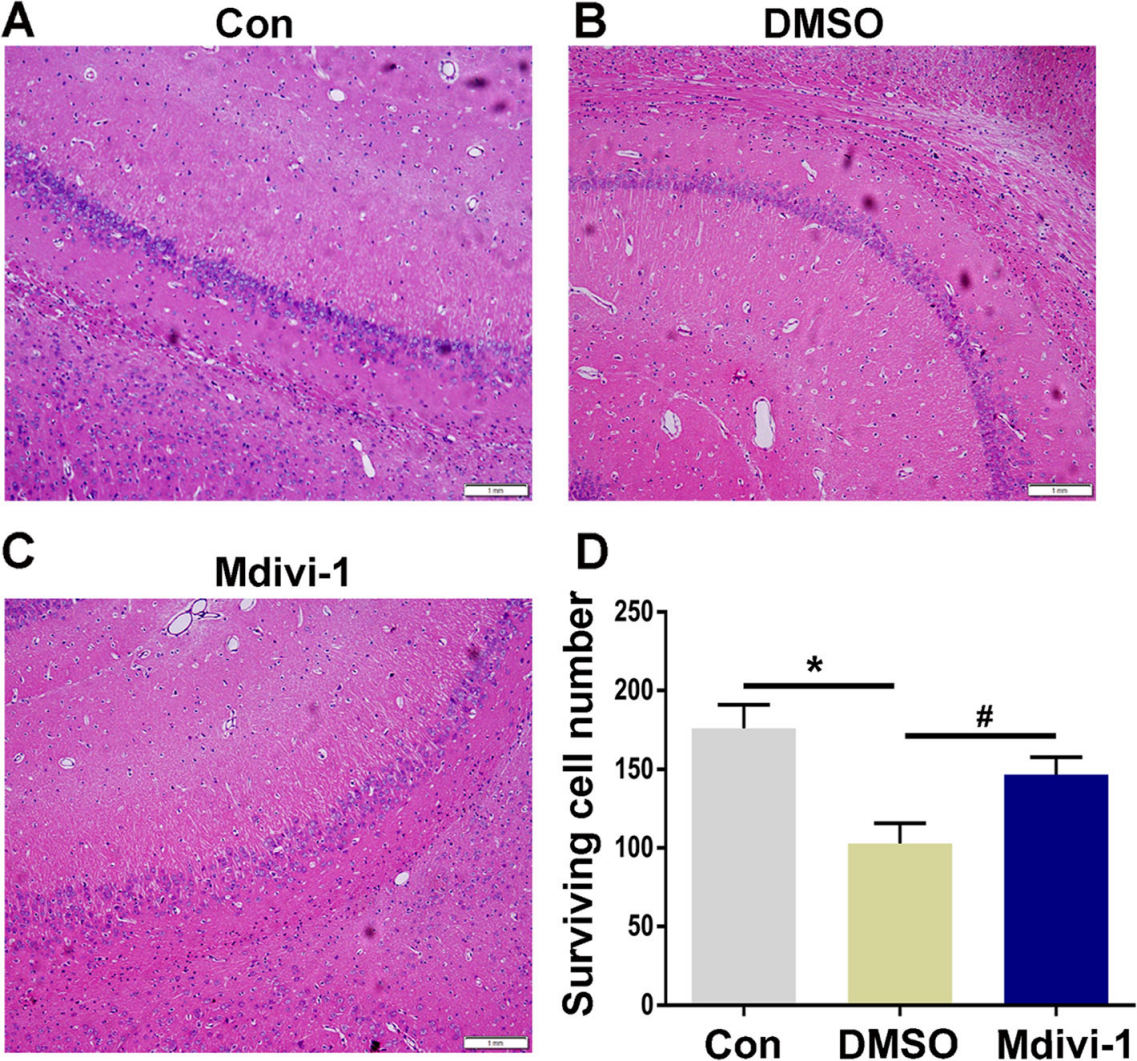
Effect of Mdivi-1 pretreatment on neuron damage after epileptic seizures. HE staining of the hippocampal CA3 pyramidal neurons at 24 h after epileptic seizures, observed under 10× magnification. **a** Control group showing normal pyramidal neurons. **b** DMSO group showing neuronal death after epileptic seizures. **c** Mdivi-1 group showing the effect of Mdivi-1 pretreatment on neuron damage after epileptic seizures. Bar = 1 mm, *n* = 5. **d** Quantitative analysis of surviving pyramidal neurons per 1-mm length of CA3 subfields in the hippocampus. *P < 0.05 compared with control, #*P* < 0.05 compared with DMSO. DMSO = pilocarpine + DMSO

Western blot analysis showed that the protein expression levels of Drp1 in the hippocampus of epileptic rats were significantly higher than those in normal control group rats, and the highest level of expression occurred 24 h after epileptic seizure onset. For this reason, 24 h after epileptic seizure was selected as the time point for measuring Drp1 expression in the hippocampus by immunohistochemistry. At 24 h after epileptic seizure, Drp1 was mainly expressed in the neuronal cell membrane of the CA1, CA3, and hilus hippocampal areas in the normal control, pilo + DMSO, and pilo + Mdivi-1 groups. In addition, we performed a semiquantitative analysis of the number of Drp1-positive cells, the number of which was significantly increased (*P* < 0.05) in the pilo + DMSO group compared with the normal control group. Moreover, the increase in number of Drp1-positive cells was significantly reduced in the pilo + Mdivi-1 group compared with the pilo + DMSO group (*P* < 0.05) (Fig. 5). Microscopic examination of the hippocampal pyramidal cells revealed wider interspaces and even cell loss in the model control rat group compared with the normal control group. However, the animals in the Mdivi-1 group showed reduced neuron damage in the hippocampal CA3 area compared with the DMSO group (Fig. 6). Thus Mdivi-1 treatment decreased cell injury in the hippocampal CA3 area of pilocarpine-induced epileptic seizure rats.

### Effects of Mdivi-1 on the expression of ENT1 in brain tissues

**Fig. 7:**
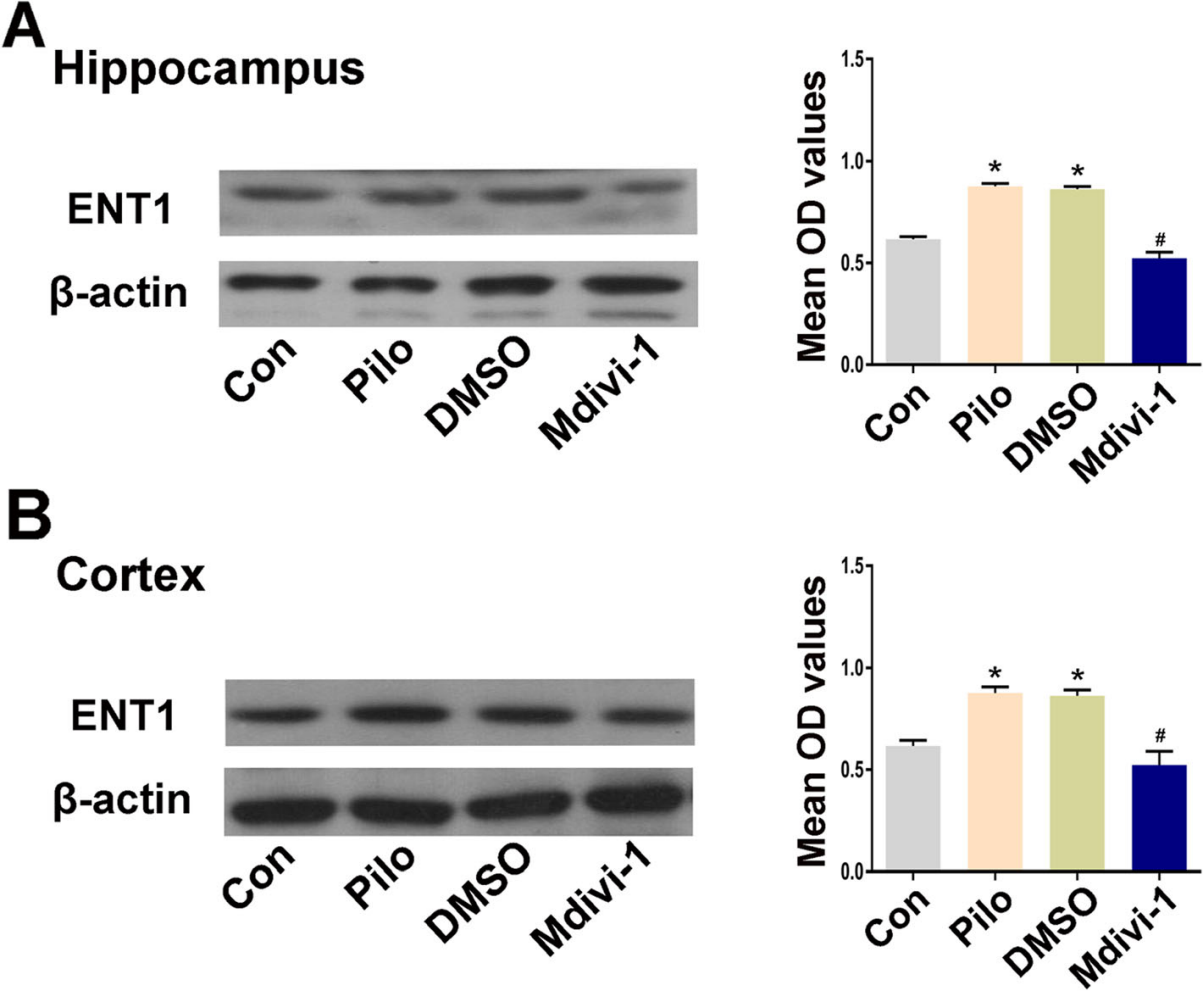
Effects of Mdivi-1 on the expression of ENT1 in brain tissues of pilocarpine-induced epileptic rats. **a**-**b** Representative images of the expression of ENT1 in the hippocampus and cortex from different groups (*n* = 5 per group) and quantification of immunoblots. Data are presented as means ± SEM and are representative of at least 3 independent repeats. *P < 0.05 compared with the Con group, ^#^*P* < 0.05 compared with the DMSO group, by one-way ANOVA followed by the LSD-*t* test

Our previous experiments demonstrated that ENT1 expression was significantly increased in the pilocarpine epileptic group 24 h after SE. To determine whether expression of ENT1 was altered by the Drp1 antagonist Mdivi-1 in pilocarpine-treated rats, western blot analysis was used to assay the expression of ENT1 in brain tissues from the different groups 24 h after SE. ENT1 was expressed at basal levels in the hippocampal tissues of rats in the normal control group. The immunoblot density ratios of ENT1 to the corresponding internal reference (β-actin) in the hippocampal tissues of rats in the normal control, pilo group, pilo + DMSO group, and pilo + Mdivi-1-treatment group were 0.56 ± 0.01, 0.81 ± 0.06, 0.79 ± 0.03, and 0.60 ± 0.09, respectively. The pilo + Mdivi-1 group exhibited decreased ENT1 expression compared with the pilo + DMSO group, and the difference was statistically significant (*P* < 0.05). The difference in ENT1 expression between the pilo and pilo + DMSO group was not statistically significant (*P* > 0.05) (Fig. 7).

In addition, in the temporal neocortex of rats, immunoblot density ratios of ENT1 to the corresponding internal reference (β-actin) in the normal control, pilo group, pilo + DMSO, and pilo + Mdivi-1-treatment group were 0.61 ± 0.02, 0.87 ± 0.02, 0.86 ± 0.02, and 0.52 ± 0.06, respectively. The pilo + Mdivi-1 group exhibited decreased ENT1 expression compared with the pilo + DMSO group, and the difference was statistically significant (*P* < 0.05). The difference in ENT1 expression between the pilo and pilo + DMSO group was not statistically significant (*P* > 0.05) (Fig. 7).

### Effects of Mdivi-1 on ultrastructural changes of mitochondria in the hippocampal CA1 region in rats from each group

**Fig. 8:**
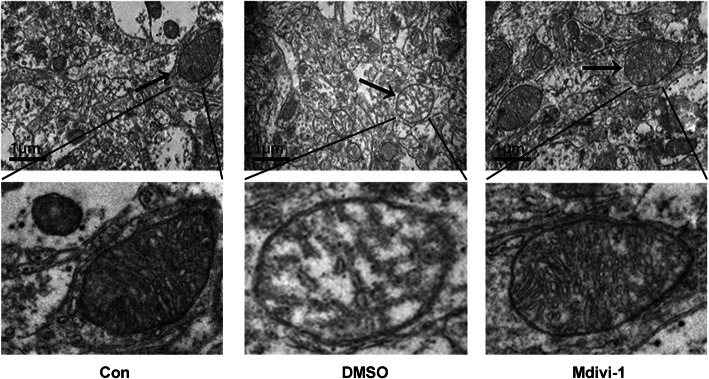
Ultrastructural changes in mitochondria in the hippocampus of epileptic rats. Ultrathin sections of hippocampus were collected from the different groups (Con, pilo + DMSO, and pilo + Mdivi-1) 24 h after pilocarpine-induced SE. Arrows mark mitochondria. *n* = 3 per group. Scale: 1 μm

Transmission electron microscopy was used to observe ultrastructural changes in neuronal mitochondria in the hippocampal CA1 region. The mitochondrial structure of rats in the normal group was intact. The neuronal mitochondria in the pilo + DMSO and pilo + Mdivi-1 groups were damaged to varying degrees. The mitochondria of neurons in the epileptic group were obviously swollen, the mitochondrial structure was destroyed, and some of the inner and outer membranes were disintegrated. In the pilo + Mdivi-1 group, neuronal mitochondria were slightly swollen, showing some division of the cristae, a decreased matrix density, and an absence of membrane disintegration (Fig. 8).

## Discussion

In the present study, we demonstrated that Drp1 expression was increased in the hippocampal and temporal neocortex tissues of SE model rats after 6 and 24 h of SE induction, and immunofluorescence analysis indicated that Drp1 was expressed mainly on neurons. Treatment with the Drp1 antagonist Mdivi-1 could suppress epileptic seizures in the pilocarpine rat model. The beneficial effects of Mdivi-1 treatment in the early phase after SE consisted of a prolonged first epileptic seizure latency and reduced number of epileptic seizures and mitochondrial damage. We also demonstrated that Mdivi-1 diminished overexpression of ENT1 and might affect ENT1 in pilocarpine-induced SE. To our knowledge, this is the first demonstration of a relationship between Drp1 and ENT1 in SE.

SE is a life-threatening emergency that is typically terminated through antiepileptic drug treatment, leading to hippocampus dysfunction typified by neurodegeneration, inflammation, and altered neurogenesis, as well as cognitive and memory deficits. An episode of extended SE is sufficient to cause chronic hippocampus dysfunction, exemplified by persistent inflammation with activation of microglia and monocyte infiltration, loss of sizable fractions of several subclasses of inhibitory interneurons, aberrant and waned neurogenesis, hippocampus-dependent cognitive and memory impairments, and chronic epilepsy [28–31]. Neurons are abnormally excited during epileptic seizures, and neuronal excitability depends primarily on energy metabolism [16, 32]. Mitochondria are located primarily outside the nucleus and are among the most important organelles in eukaryotes, mainly functioning to provide energy via ATP production [33]. Extended SE can lead to changes in mitochondrial ultrastructure and increased free radical content, resulting in imbalanced mitochondrial homeostasis [34]. Several studies have noted that epileptic seizures not only affect mitochondrial structure and function but also increase neuronal excitability, thus forming a vicious circle [8, 35, 36]. Similarly, ultrastructural changes in hippocampal neurons have been observed following epileptic seizures, indicating severe mitochondrial damage.

Drp1 is a key protein that regulates mitochondrial division, mainly in the form of cytoplasmic polymers [37]. Drp1 mediates mitochondrial division while interacting with the apoptosis-related proteins Bax and Bak, which together result in neuronal damage [38]. In addition, Drp1 dysfunction can impair the intrinsic biological energy function of axons and mitochondria, which can lead to axonal dysfunction [39]. Our experimental study showed that Drp1 protein expression increased within 6 h after epileptic seizure, reaching peak expression at 24 h and then slowly declining. After epileptic seizure, the number of Drp1-positive cells also increased, and Drp1 was expressed in hippocampal neurons. Drp1 appears to be a promising target for the treatment of epilepsy. Epileptic seizures have been reported to increase astrocyte apoptosis in the hippocampus, which may be related to Drp1-mediated mitochondrial dynamics [40]. Drp1 can modulate synaptic plasticity by regulating the growth of dendrites in cerebellar Purkinje cells [41]. Recently, studies have shown that Drp1 expression increases after epileptic seizures in rats and that inhibition of mitochondrial division can alleviate neuronal damage after epilepsy [10], an observation that is consistent with some of our experimental results [42]. Three cases of epileptic seizures upon mitochondrial-related protein deletion were reported in two families, and abnormal Drp1 expression was detected by immunofluorescence staining [43]. A case of intractable epilepsy caused by a DNM1L-associated mitochondrial fission disorder has been reported, which demonstrates that Drp1 is involved in the development of epilepsy.

Mdivi-1 is a cell-permeable ketone compound, and in vitro studies have shown that it can block dynamin 1 (Dnm1) by allosterically regulating the ATP content in cells. Mdivi-1 further inhibits cell apoptosis by inhibiting mitochondrial outer membrane permeabilization [44]. Mdivi-1 is a selective inhibitor of Drp1 and regulates Drp1 function mainly by inhibiting its GTPase activity [45]. The mechanism underlying this phenomenon may be related to the release and activation of apoptosis-related factors via the inhibited release of CytC [11]. Our study showed that the latency of seizures in epileptic rats was prolonged by treatment with Mdivi-1, and the expression of Drp1 was lower in the hippocampal and temporal lobe cortex tissues of rats in the Mdivi-1 group than the epileptic group at the same timepoint. This observation suggests that Mdivi-1 can reduce epileptic seizures by inhibiting Drp1. Studies have found that Mdivi-1 inhibits mitochondrial and neuronal apoptosis, and it has a protective effect on neurons [46, 47]. Other studies have found that Mdivi-1 can significantly reduce oxidative stress, increase the activity of superoxide dismutase (SOD), and reduce the expression of CytC and Caspase 3, thus protecting the hippocampus [38].

The main function of ENT1 is to regulate the levels of adenosine. ENT1 is expressed in the granule cells of the hippocampus, vertebral cells, and DG in rats, which are rich in adenosine A receptors [48]. In astrocytes, adenosine is formed by the breakdown of ATP, whereas ATP is released by astrocytes, and neurons can sense extracellular adenosine [49]. In vitro patch clamp experiments in rat hippocampal slices have shown that ENT1 inhibition can reduce the frequency of neuronal action potentials while also reducing the amplitude of miniature excitatory postsynaptic currents, which has a protective effect on neurons after epileptic seizures [22]. Therefore, we chose to examine ENT1 expression in the Drp1-specific Mdivi-1 suppression group, revealing significantly lower expression than in the epileptic group. The Drp1-mediated imbalance in mitochondrial dynamics affects the production of ATP, and ATP synthesis disorders can lead to abnormal expression of extracellular adenosine by inhibiting Drp1, which ultimately affects ENT1 expression; however, the specific mechanism underlying this phenomenon requires further study.

## Conclusion

In summary, the present study provides evidence for the neuroprotective effect of Mdivi-1 in mitochondrial morphology and neuron damage in the pilocarpine-induced SE rat model. Mdivi-1 attenuates the severity of seizures regulating ENT1 expression, possibly via a mechanism in which the Drp1-mediated imbalance in mitochondrial dynamics disrupts ATP production, leading to decreased adenosine levels and thereby altering the expression and function of ENT1. The specific mechanism, however, requires further study.

## Supplementary information

**Additional file 1.**

## Data Availability

The datasets used and/or analysed during the current study are available from the corresponding author on reasonable request.

## Abbreviations

Drp1: Dynamic-related protein 1
ENT1: Equilibrative nucleoside transporter 1
SE: Status Epilepticus
AEDs: Antiepileptic drugs
CNS: Central nervous system
RIPA: Radioimmune precipitation buffer
HRP: Horseradish peroxidase
HE: Hematoxylin and eosin
MAP 2: Microtubule-associated protein 2
GFAP: Glial fibrillary acidic protein
ANOVA: One-way analysis of variance
Dnm1: Dynamin 1
CytC: Cytochrome C
SOD: Superoxide dismutase
