# Supplementary figures and images for: Dynamic-related protein 1 inhibitor eases epileptic seizures and can regulate equilibrative nucleoside transporter 1 expression
Source: BMC Neurol. 2020 Sep 22;20:353. doi: 10.1186/s12883-020-01921-y (PMC7507736; doi:10.1186/s12883-020-01921-y)

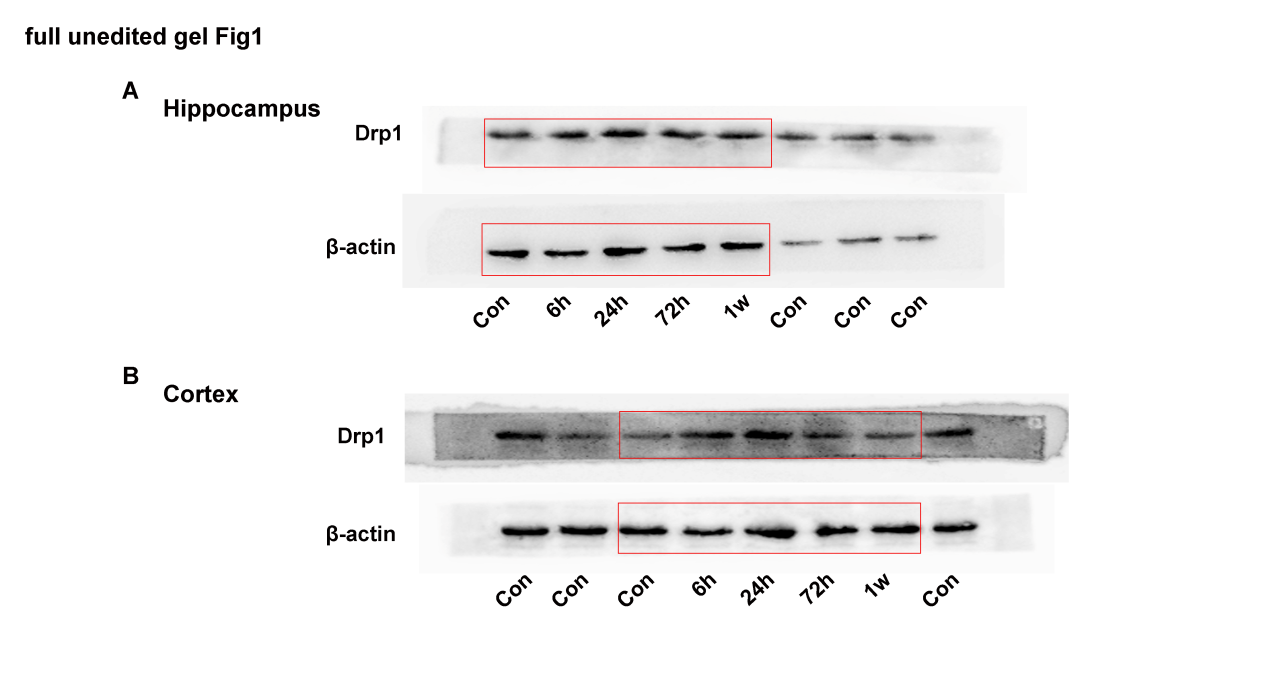


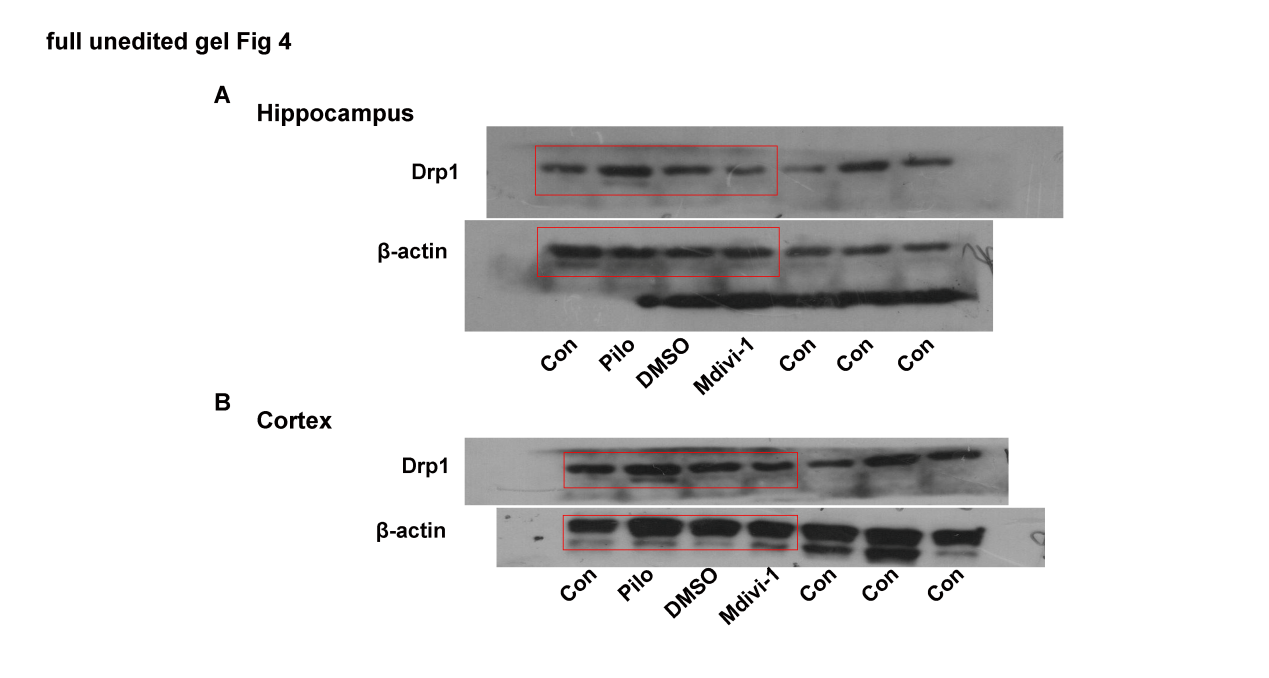


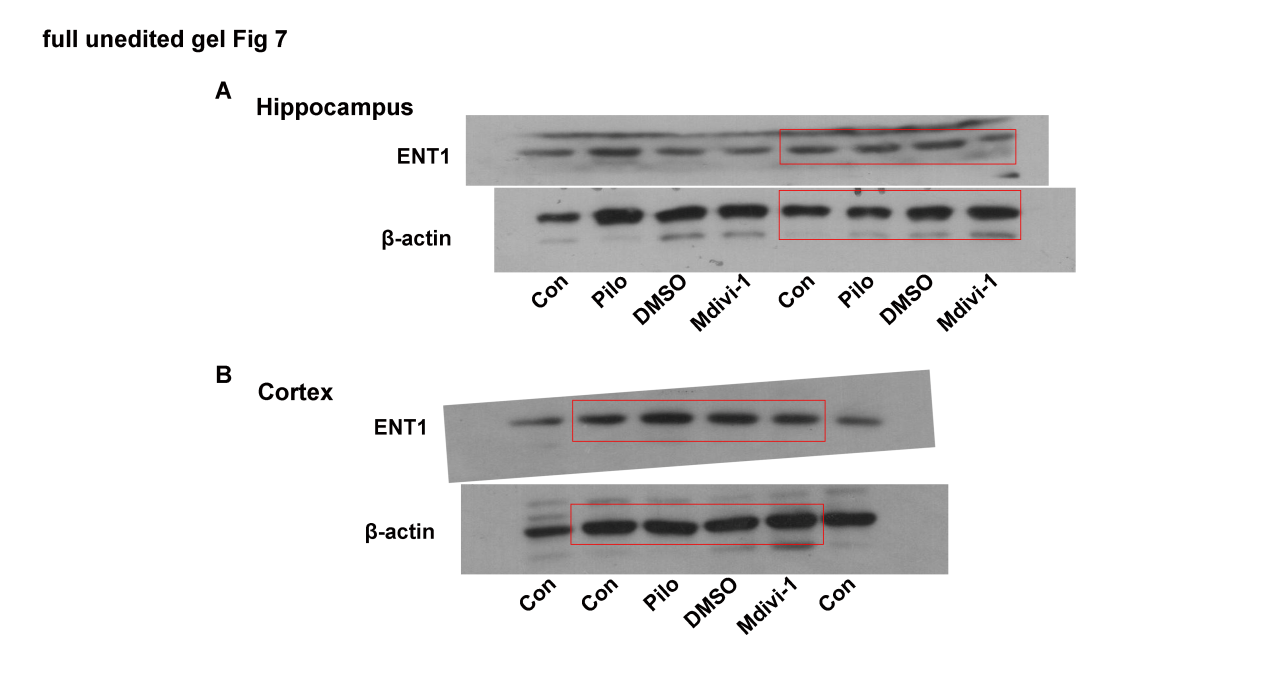

Supplement: Supplementary file 1 — Additional file 1. [file 12883_2020_1921_MOESM1_ESM.docx]
